# Multimodal evaluation of blood-brain barrier opening in mice in response to low-intensity ultrasound and a claudin-5 binder

**DOI:** 10.7150/ntno.95146

**Published:** 2024-04-23

**Authors:** Liyu Chen, Jae Song, Gina Richter-Stretton, Wendy Lee, Pranesh Padmanabhan, Jürgen Götz

**Affiliations:** Clem Jones Centre for Ageing Dementia Research (CJCADR), Queensland Brain Institute (QBI), The University of Queensland, Brisbane QLD 4072, Australia.

**Keywords:** blood-brain barrier, broadband emission, claudin-5, focused ultrasound, immunoglobulins

## Abstract

**Background:** The blood-brain barrier (BBB) is a major bottleneck in delivering therapeutics to the brain. Treatment strategies to transiently open this barrier include focused ultrasound combined with intravenously injected microbubbles (FUS^+MB^) and targeting of molecules that regulate BBB permeability.

**Methods:** Here, we investigated BBB opening mediated by the claudin-5 binder cCPEm (a microorganismal toxin in a truncated form) and FUS^+MB^ at a centre frequency of 1 MHz, assessing dextran uptake, broadband emission, and endogenous immunoglobulin G (IgG) extravasation.

**Results:** FUS^+MB^-induced BBB opening was detectable at a pressure ≥0.35 MPa when assessed for leakage of 10 and 70 kDa dextran, and at ≥0.2 MPa for uptake of endogenous IgG. Treating mice with 20 mg/kg cCPEm failed to open the BBB, and pre-treatment with cCPEm followed by FUS^+MB^ at 0.2 and 0.3 MPa did not overtly increase BBB opening compared to FUS^+MB^ alone. Using passive cavitation detection (PCD), we found that broadband emission correlated with the peak negative pressure (PNP) and dextran leakage, indicating the possibility of using broadband emission for developing a feedback controller to monitor BBB opening.

**Conclusions:** Together, our study highlights the challenges in developing combinatorial approaches to open the BBB and presents an additional IgG-based histological detection method for BBB opening.

## Introduction

The blood-brain barier (BBB) is a dynamic semipermeable interface that regulates the bidirectional transport of molecules between the circulation and the central nervous system (CNS), thereby posing a formidable hurdle for drug delivery to the brain 1. A defining feature of the BBB is its tightly packed monolayer of non-fenestrated brain endothelial cells with low pinocytic activity, i.e., the limited internalisation of liquids and solutes from the surrounding space via small pinocytic vesicles. The endothelial cells interact with other cell-types and the basement membrane to form the neurovascular unit (NVU) 2. Brain endothelial cells are closely connected through junctional protein complexes, including tight junctions (TJs) and adherens junctions (AJs), which restrict the paracellular diffusion of molecules in a size- and charge-selective manner 3.

To improve paracellular drug delivery across the BBB, the highly expressed TJ protein, claudin-5, has previously been targeted by either transiently knocking down claudin-5 or by delivering claudin-5-specific binders, including cCPEm as a truncated form of clostridium perfringens enterotoxin (an enterotoxin being a protein exotoxin released by a microorganism and causing gastrointestinal symptoms) 4-7. This mutant protein has a nanomolar binding affinity for the second extracellular loop domain, ECL2, of claudin-5 7. In murine brain vascular endothelial bEND.3 cells, cCPEm has been shown to cause reduced transendothelial electrical resistance (TEER), a readout of BBB permeability 8. In zebrafish larvae, intravenous administration of cCPEm enhanced permeation of 10 kDa dextran for up to 3 h after administration 8. Given this evidence that cCPEm has the potential of regulating the permeability of BBB, the question arises how this modality compares to other BBB-weakening strategies, and how it would operate in mice.

Low-intensity focused ultrasound (FUS) in conjunction with intravenously injected, biologically inert gas-filled microbubbles (FUS^+MB^) is an emerging strategy to transiently and locally open the BBB for enhanced cerebral drug delivery to treat human brain disorders 3. In response to the ultrasound pulses, the circulating MBs oscillate, exerting mechanical stress on brain endothelial cells and leading to a separation of TJ strands, an increased number of vesicles and vacuoles, as well as a reduced expression of drug transporters such as P-glycoprotein, thereby enhancing both paracellular and transcytoplasmic transport 9-11. FUS^+MB^ has been explored extensively in many experimental animal species and has already entered the clinical trial space for several therapeutic indications 12, 13. Given that we have previously demonstrated in human claudin-5-expressing epithelial MDCKII cells that pre-incubation with cCPEm improves barrier opening in response to FUS^+MB^, we here assessed whether FUS^+MB^ and cCPEm, either alone or in combination, cause leakage of fluorescently labelled dextrans sized 10 and 70 kDa *in vivo*, using wild-type mice 14. We also monitored the uptake of endogenous immunoglobulins (IgG), which we found to be a sensitive read-out for detecting BBB opening at ultrasound pressures below those achieving dextran leakage.

We further monitored microbubble activity by passive cavitation detection (PCD), extracting harmonic and broadband emissions 15, 16. We then evaluated the correlation of harmonic and broadband emission with the peak negative pressure (PNP), a key acoustic parameter in achieving BBB opening. In addition, the correlation of harmonic and broadband emissions was evaluated with the mean fluorescence intensity (MFI) obtained for 10 and 70 kDa dextran leakage.

## Results

### Study design and characterisation of custom-designed microbubbles

**Figure 1:**
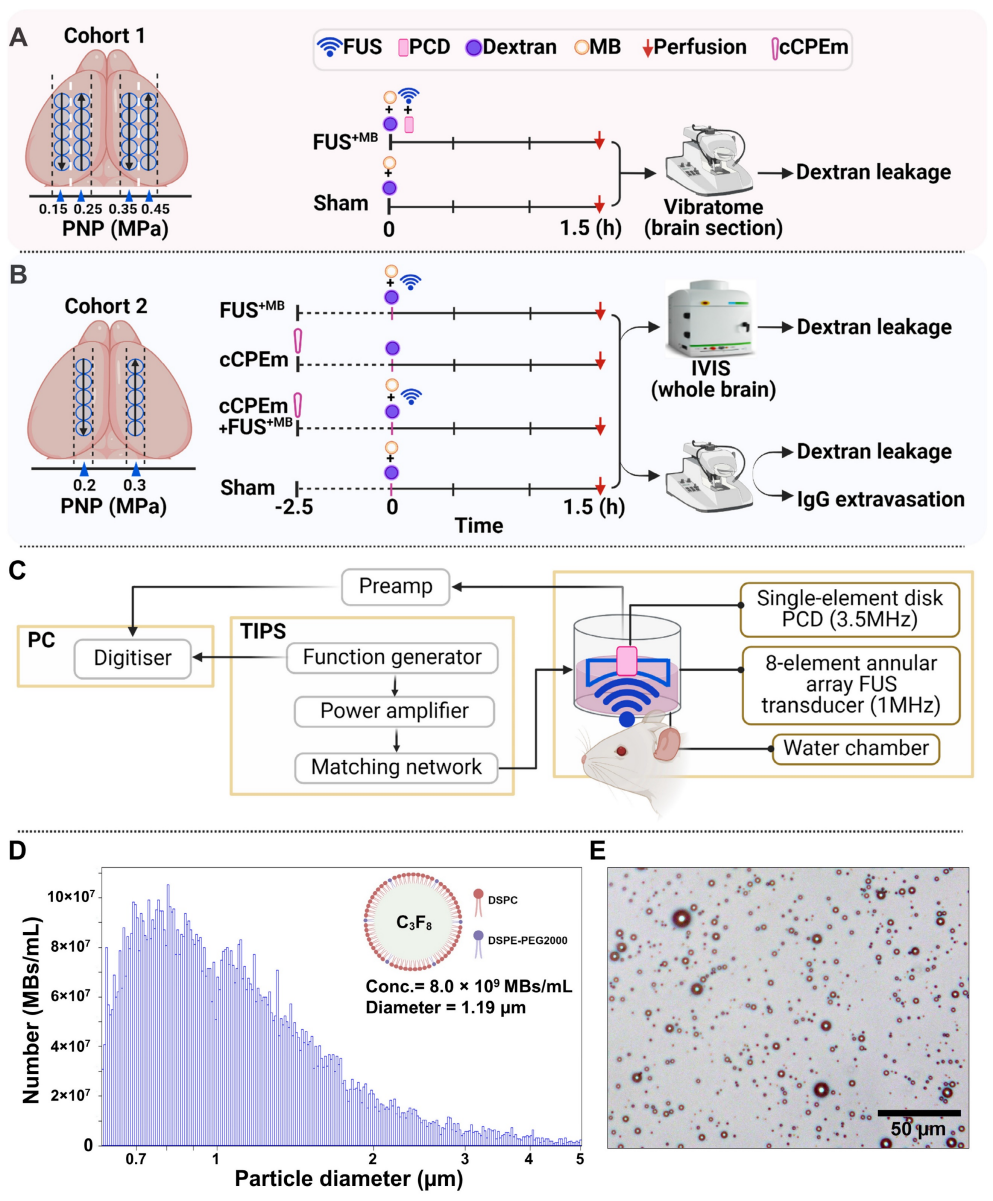
** Study design, ultrasound system setup and microbubble characterisation. (A,B)** Experimental design involving two study cohorts (created with BioRender.com). (**A**) Cohort 1 was designed to explore the focused ultrasound plus microbubble (FUS^+MB^)-induced BBB opening threshold, investigating peak negative pressures (PNPs) ranging from 0.15 to 0.45 MPa. (**B**) Cohort 2 was designed to compare the efficacy of a single cCPEm treatment, only FUS^+MB^ treatment, and pre-treatment with cCPEm prior to FUS^+MB^ (cCPEm+FUS^+MB^). The transducer was moved as indicated by the black arrows. BBB opening was visualized on brain sections or in the IVIS system as indicated, upon co-injecting two dextrans (FD10, 10 kDa, together with TAMRA-D70, 70 kDa). (**C**) Ultrasound setup composed of a TIPS system operated from a PC with a digitiser, and a PCD to detect acoustic signatures (harmonic and broadband emissions) in response to FUS^+MB^. (**D**) Using a Coulter counter, the concentration and mean diameter of the customised microbubbles were determined as 8.0 × 10^9^ MBs/mL and 1.19 µm, respectively (3 independent experiments), (**E**) with the microbubbles displaying a multi-dispersed size distribution. Scale bar = 50 µm.

We used two cohorts of wild-type mice. In cohort 1, low-intensity focused ultrasound was explored over a range of PNPs in the presence of intravenously injected, biologically inert gas-filled microbubbles (FUS^+MB^) (Fig. 1A). Fluorescently labelled dextrans of two sizes, FD10 (10 kDa) and TAMRA-D70 (70 kDa), were injected to determine brain leakage. As a negative sham control, mice received dextrans and microbubbles without ultrasound pulses. The mice were sacrificed 1.5 hours after the treatment guided by work that had demonstrated peak BBB opening one hour after sonication 17. The brains were dissected, and BBB opening was determined on whole brain sections by examining the brain leakage of FD10 and TAMRA-D70. In cohort 2, we tested cCPEm, a claudin-5-binder that has been shown *in vitro* to both weaken the BBB integrity on its own 7, 18 and to lower the PNP threshold required for FUS^+MB^-mediated barrier opening 14. We therefore assessed cCPEm on its own and in combination with FUS^+MB^ in mice, including a sham and FUS^+MB^ group (Fig. 1B). Of note, cCPEm was administered 2.5 hours before ultrasound sonication, guided by a small pilot time-course experiment we had conducted to determine the optimal timepoint for pre-treating the mice with cCPEm (data not shown). In the pilot, we had observed the strongest permeability for FD10 2 hours after cCPEm treatment, and a slightly weaker signal 3 hours after administration. The mice in cohort 2 were injected with dextrans (and sonicated) at time-point 0 and sacrificed 1.5 hours later, resulting in a procedure lasting 4 hours in total. Besides determining dextran leakage on brain sections, we also analysed dextran intensity in whole brain semi-quantitatively, using a Bruker *In Vivo* MS FX Pro optical imaging system (IVIS), and stained sections for endogenous IgG extravasation. The 1 MHz TIPS system with its 8-element annular array transducer was used for the FUS^+MB^ treatment, and a PCD was employed to simultaneously capture the acoustic signature of the microbubbles in response to the ultrasound delivery (without functioning as a feedback control) (Fig. 1C).

As a quality control measure, we determined the physical properties of our custom-designed MBs as done by us previously 19, before employing them in the FUS^+MB^ treatments. Using a Coulter counter, we found a mean concentration of 8.0 ×10^9^ MBs/mL and a mean diameter of 1.19 μm (Fig. 1D), which represent values similar to what has been reported previously for commercially available microbubbles such as Definity 20, 21. Under an epifluorescence microscope, the MBs displayed a multi-dispersed size distribution (Fig. 1E).

### FUS^+MB^-mediated dextran leakage observed at higher peak negative pressures

The PNP is a critical parameter in FUS^+MB^-mediated BBB opening. Guided by previous studies, in cohort 1, we explored PNPs of 0.15, 0.25, 0.35 and 0.45 MPa, anticipating dextran leakage at the higher pressures. In the interest of reducing the number of mice in our study, the four PNPs were applied in four rows each, by delivering five sonication spots per row for the two lower pressures to the left hemisphere, and similarly for the two higher pressures to the right hemisphere (Fig. 1A). Prior to the treatment, the mice (n=4) were positioned in a stereotaxic frame and coupled with coupling gel to the TIPS ultrasound transducer, that was operated with a personal computer (PC)-triggered, radio frequency (RF)-amplified function generator. The two dextrans, FD10 and TAMRA-D70 were mixed and *i.v.* injected together. Immediately thereafter, the microbubbles were bolus-injected by hand. To deliver the sonications, the TIPS transducer was moved as indicated by arrows (Fig. 1A), starting from the left hemisphere, by means of a programmable motorized positioning system, with five 6 second-sonication spots per PNP spaced 1.5 mm apart. Given the short half-life of microbubbles in the circulation due to ultrasound-induced cavitation, they were replenished after the first two rows of sonication by injecting a second aliquot (Fig. 1A).

**Figure 2:**
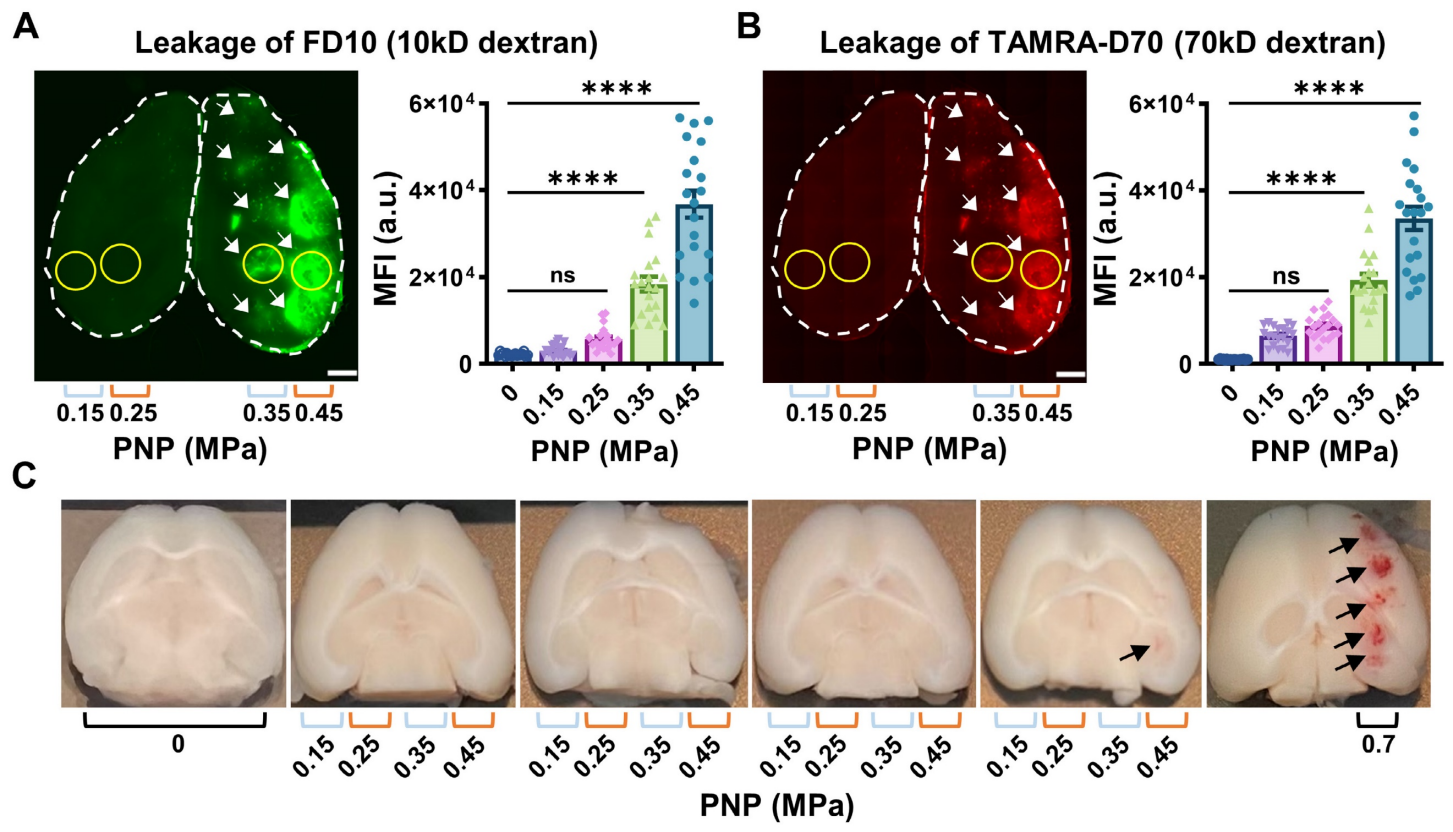
** Leakage of two types of dextran following ultrasound treatment and safety analysis.** (**A,B**) Representative 100 μm thick horizontal brain slices displaying leakage of the fluorescently tagged dextrans FD10 (10 kDa, green, **A**) and TAMRA-D70 (70 kDa, red, **B**). The dashed lines highlight the boundaries of the left and right hemisphere. Mean fluorescence intensity (MFI) given in arbitrary units (a.u.) for dextran leakage was quantified using ImageJ. For each PNP, a region of interest (ROI, yellow circle), corresponding to the sonication spot with the MFI for a given section was chosen, quantifying five sections per mouse. Given the limited targeting precision of the FUS setting and differences in brain anatomy between mice, the maximal number of spots detected per section were 5. Scale bar = 1 mm (N=4 mice from two independent experiments, with a total of 20 dots representing the MFI of 20 sonication spots from 20 tissue slices, 5 slices per mouse, mean ± SEM; one-way ANOVA Tukey's multiple comparisons analysis, ns: not significant, p****<0.0001). (**C**) Representative images of mouse tissue displaying moderate bleeding at 0.45 MPa (only one spot out of twenty, see arrow) and strong bleeding at 0.7 MPa (five spots, arrows).

We assessed BBB opening 1.5 hours after the treatment by determining FD10 and TAMRA-D70 leakage in brain sections using an upright epifluorescence Axio Imager Azure microscope. For each PNP, a region of interest (Fig. 2A,B, ROI, yellow circle), corresponding to the sonication spot with the maximal fluorescence signal intensity for a given section was chosen, quantifying five sections per mouse. Reflecting the targeting precision of the FUS setting and differences in brain anatomy across mice, several sections had less than 5 sonication spots (Table 1). At 0.45 MPa, we observed substantial dextran leakage for both sizes (*p<0.0001*, compared with sham), moderate leakage at 0.35 MPa, and no detectable leakage at 0.25 and 0.15 MPa (Fig. 2A,B, Table 1). The threshold for FUS^+MB^-mediated BBB opening assessed by dextran leakage was between 0.25 and 0.35 MPa for our sonication setup, with an absence of BBB opening at the lower pressures. This observation was in line with that reported by other groups 22, 23.

### Gross anatomical analysis in correlation with passive cavitation detection

A read-out of tissue damage induced by FUS^+MB^ is the presence of petechiae, i.e., the extravasation of erythrocytes at endothelial junctions. No extravasation was observed for any of the explored PNPs, except for one mouse, for which we observed subtle bleeding for one sonication spot at the highest PNP of 0.45 MPa only (Fig. 2C, arrow). For reference, we also included mice (n=4) in which we delivered a row of five sonications at 0.7 MPa, consistently revealing intense bleeding and thereby clearly demarcating the five sonication spots (Fig. 2C, arrows). A PNP of 0.7 MPa was deemed non-suitable for safe FUS^+MB^-mediated BBB opening in our setup.

**Figure 3:**
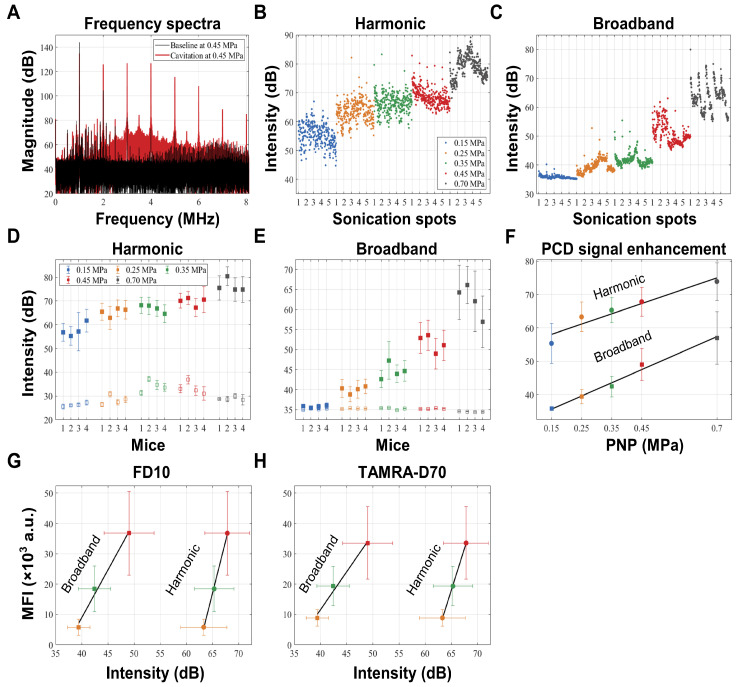
** Passive cavitation detection and correlation with pressure and dextran leakage.** (**A**) A representative frequency spectrum of the raw PCD signal obtained at 0.45 MPa in the absence of microbubbles (in black), defining baseline levels, and in the presence of microbubbles (in red), showing harmonic and broadband emission above baseline. (**B,C**) Absolute intensities of harmonic and broadband emissions obtained from individual PCD signals plotted in the order of the sonications. (**D,E**) Average harmonic and broadband intensities due to microbubble cavitation (closed square) and at baseline (open square) for each PNP from the four mice plotted with standard deviation, revealing that the microbubbles effectively cavitated at all applied PNPs, and that the procedure was reproducible for all animals. Harmonic and broadband intensities increased from 0.15 to 0.7 MPa, but broadband intensity increased more linearly and was more clearly separated for the different PNPs. **(F)** Both harmonic (r^2^=0.92; p=0.01) and broadband emissions (r^2^=0.99; p=0.0007) are strongly correlated with the PNP. (**G,H**) Harmonic emission is correlated with FD10 leakage (r^2^=0.99; p=0.069) and TAMRA-D70 leakage (r^2^=0.99; p=0.081). Broadband emission is correlated with FD10 leakage (r^2^=0.99; p=0.032) and TAMRA-D70 leakage (r^2^=0.99; p=0.0194), displayed as MFI in arbitrary units (a.u.).

Given the ongoing efforts in the field of using cavitation as a means to monitor safe BBB opening, we measured cavitation activities during the FUS^+MB^ treatment, using a custom-built PCD system 24-26. Cavitation activities were measured via two acoustic components, harmonic emissions and broadband emissions. We aimed to assess a potential correlation of this signature with BBB opening through a series of analyses: i) The raw frequency spectrum was calculated using Fourier transform, from which the 4^th^ and 5^th^ harmonics and broadband emissions ranging from 3 to 5 MHz (excluding the harmonics) were extracted (Fig. 3A), ii) the intensities of both acoustic components were calculated from individual pulses (Fig. 3B,C), iii) then, for each PNP averaging was applied to all 5 sonication spots per mouse (Fig. 3D,E), and finally, iv) average intensities of both emissions were used to determine a correlation of the PCD signal both with the PNP (Fig. 3F) and with the leakage of the two dextrans, FD10 and TAMRA-D70, displayed as MFI (Fig. 3G,H).

We firstly found that the average intensities of harmonic and broadband emissions measured for the four sonication rows (0.15 - 0.45 MPa) as well as one additional row separately treated at 0.7 MPa within each group were comparable, suggesting a consistency of microbubble delivery and their response to FUS^+MB^ (Fig. 3D,E). At 0.15 MPa, harmonic emissions in the presence of microbubbles developed with an intensity enhancement of 29 dB from baseline levels (Table 2), whereas in contrast, little broadband emission was detected at this low pressure. With the PNP increasing to 0.7 MPa, both harmonic and broadband intensities continued to increase. Indeed, both harmonic emission (r^2^=0.92; p=0.01) and broadband emission (r^2^=0.99; p=0.0007) were found to be strongly correlated with the PNP (Fig. 3F). Notably, harmonic and broadband intensities also correlated with the MFI of FD10 and TAMRA-D70 leakage for PNPs ranging from 0.25 to 0.45 MPa. Harmonic emission was found to be correlated with FD10 leakage (r^2^=0.99; p=0.069) and TAMRA-D70 leakage (r^2^=0.99; p=0.081), and broadband emission with FD10 leakage (r^2^=0.99; p=0.032) and TAMRA-D70 leakage (r^2^=0.99; p=0.0194) (Fig. 3G,H). Of note, for these calculations, absolute cavitation intensities were used, i.e., baseline levels were not subtracted. For baseline-subtracted harmonic emissions, the correlation was poor due to variability in the baseline of harmonic emission (Fig. 3D), possibly caused by imperfect coupling or variability in tissue non-linearity 27, as can also be inferred from Table 2.

### Pre-treatment with cCPEm does not augment FUS^+MB^-mediated dextran leakage

Having established in cohort 1 that FUS^+MB^-mediated BBB opening occurs at 0.35 and 0.45 MPa, we next explored the claudin-5-binder cCPEm at 20 mg/kg *in vivo*, given we had previously shown that this binder weakens the barrier in a cell culture system and moreover lowers the PNP required for FUS^+MB^ to open the barrier as measured by TEER measurement and dextran leakage 14.

**Figure 4:**
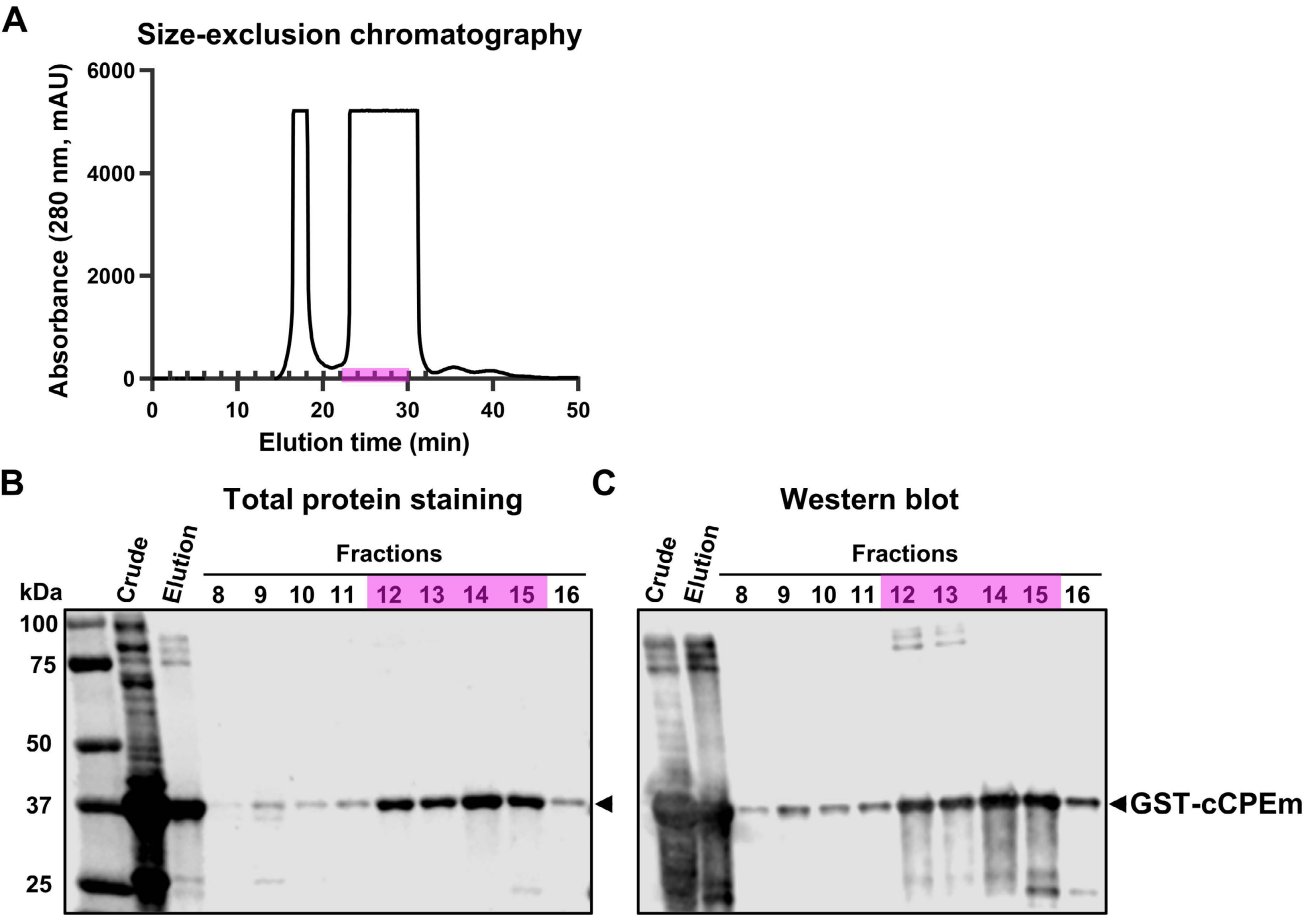
** Characterization of cCPEm fused with a GST tag. (A)** Size-exclusion chromatography purification of the elution fraction collected by affinity chromatography using a GST-column. (**B,C**) Total protein staining and western blot analysis of the crude lysate and elution fraction as well as fractions collected by size-exclusion chromatography, using an anti-GST antibody. Fractions 12-15 (highlighted in pink) were pooled and used in our *in vivo* study.

The cCPEm protein was generated and purified in-house using a two-step strategy involving affinity chromatography and size-exclusion chromatography (Fig. 4A). Given that cCPEm was designed with a GST-tag, an anti-GST antibody was used to demonstrate the purity of the cCPEm preparation by immunoblotting (Fig. 4B,C).

**Figure 5:**
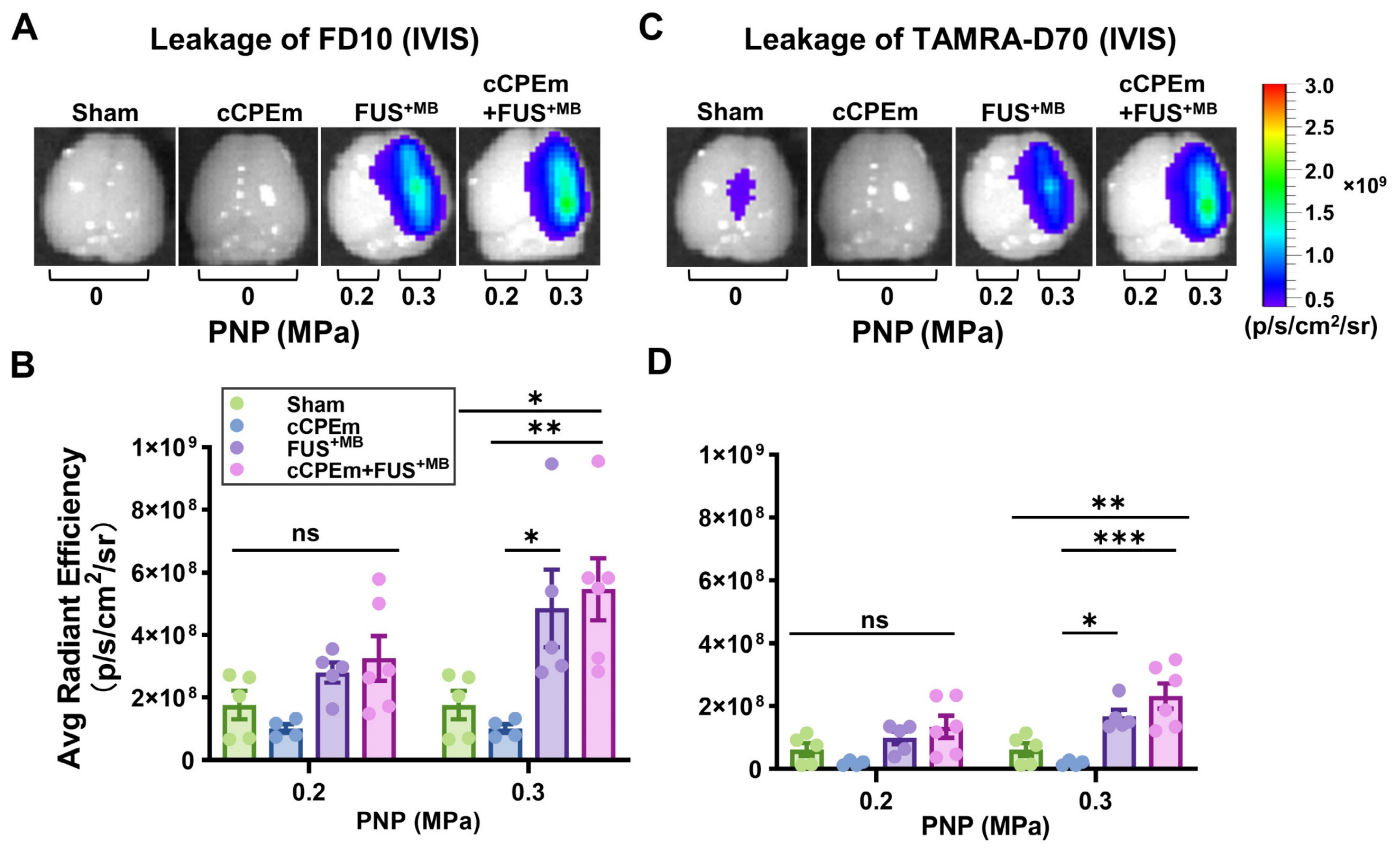
** Treatment with cCPEm neither opens the blood-brain barrier nor augments FUS^+MB^-mediated dextran leakage.** Representative image of whole brains of treated mice and quantification of the fluorescence intensity for 10 kDa (**A,B**) and 70 kDa dextran (**C,D**) using the Bruker *In Vivo* MS FX Pro optimal imaging system (IVIS). No significant fluorescence intensity was observed for mice treated with cCPEm and the sham group. Pre-treatment with cCPEm at 20 mg/kg 2.5 hours prior to FUS^+MB^ treatment did not significantly increase leakage of FD10 and TAMRA-D70 compared to FUS^+MB^ alone (n≥4, each bar dot representing the average fluorescence intensity (avg radiant efficiency) obtained from one brain hemisphere, mean ± SEM; Two-way ANOVA with Tukey's multiple comparisons tests, ns: not significant, *^✱^p*<0.05*, ^✱✱^*p<0.01* and ^✱✱✱^p*<0.001).

**Figure 6:**
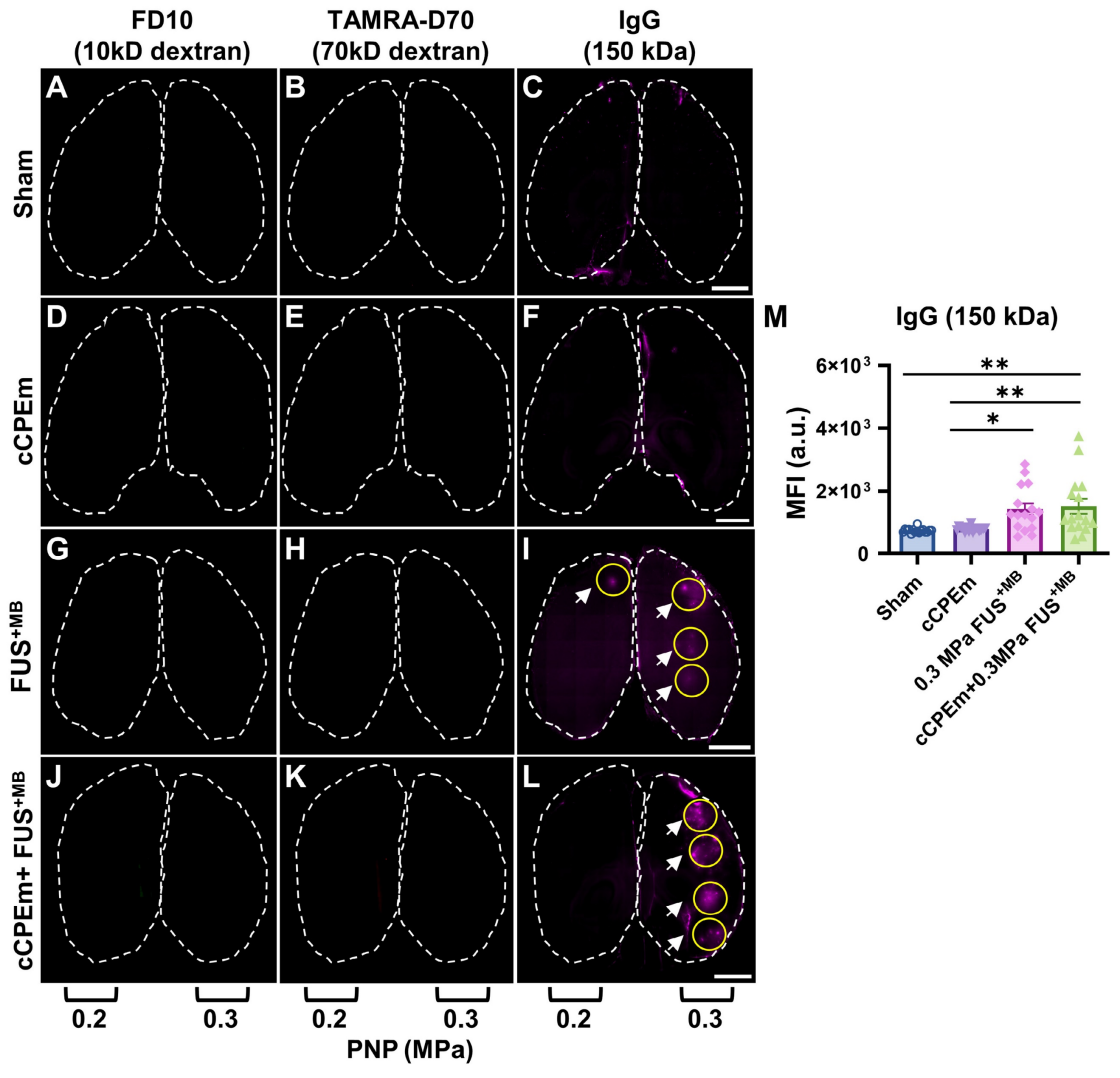
** Endogenous immunoglobulin provides a sensitive readout for BBB opening, complementing dextran uptake measurements.** Representative horizontal brain slices (100 μm thick) displaying leakage of FD10 (green, **A, D, G and J**), TAMRA-D70 (red, **B, E, H and K**) and endogenous IgG (purple, **C, F, I and L**). Dashed lines highlight the boundaries of the left and right hemisphere. Dextran leakage was not achieved at the lower acoustic pressures (0.2 and 0.3 MPa) (**G,H, J,K**). However, endogenous IgG leakage was detectable for FUS^+MB^ and cCPEm + FUS^+MB^ at 0.3 MPa (**I, L,** white arrow) and to some extent at 0.2 MPa (**I**, white arrow). (**M**) MFI given in a.u. for endogenous IgG leakage quantified using ImageJ for the sonicated region, which was subtracted from the sham control. A region of interest (ROI, yellow circle), corresponding to one sonication spot, was selected based on the maximal fluorescence signal of each tissue slice. Scale bar = 2 mm. (N≥4 from four independent experiments with a total of 16 bar dots representing the MFI of 16 sonication spots from 16 tissue slices, 4 slices per mouse, mean ± SEM; one-way ANOVA with Tukey's multiple comparisons tests, *^✱^p*<0.05* and ^✱✱^p*<0.01).

To determine whether cCPEm would achieve BBB opening in mice (reflecting barrier opening *in vitro*), we systemically administered 20 mg/kg cCPEm, which is a concentration around half of what has been used previously around 72 hours post fertilization in zebrafish larvae to achieve BBB opening 8. However, when we analysed the cCPEm-treated mice 4 hours after the infusion of cCPEm (and 1.5 hours after dextran injection) in the Bruker *In Vivo* MS FX Pro optical imaging system for whole brain fluorescence, we neither detected FD10 nor TAMRA-D70 leakage, with the quantified signal being comparable to that for the sham control (Fig. 5A-D). We next imaged dextran leakage in brain sections using the Axio Imager Azure microscope as done for cohort 1 (Fig. 2A,B), including a sham control (Fig. 6A,B), and again found no dextran leakage after cCPEm treatment, using sham as control (Fig. 6D,E).

We next asked whether pre-treatment with cCPEm followed by FUS^+MB^ would achieve dextran leakage, again guided by our earlier *in vitro* studies 14. Given that in cohort 1 we had identified the PNP threshold for FUS^+MB^-mediated BBB opening between 0.25 and 0.35 MPa (Fig. 2A,B), for cohort 2, we opted for 0.2 and 0.3 MPa as parameters for the combination treatment. 0.3 MPa is close to the FUS^+MB^-mediated BBB opening threshold whereas 0.2 MPa is lower. We anticipated that if cCPEm were able to facilitate FUS^+MB^-mediated BBB opening, this PNP range would be ideal to explore. As expected, there was no overt leakage of either of the two dextrans at 0.2 MPa, but there was also no leakage at 0.3 MPa, as revealed by whole brain imaging and examining brain sections (Fig. 5A-D, Fig. 6G,H). This narrowed down the threshold for FUS^+MB^-mediated BBB opening detected by FD10 or TAMRA-D70 uptake to between 0.3 and 0.35 MPa. We therefore injected mice with 20 mg/kg cCPEm 2.5 hours prior to FUS^+MB^ treatment at 0.2 and 0.3 MPa and sacrificed them 1.5 hours post FUS^+MB^ treatment for analysis. However, different from the previously reported *in vitro* findings, for the conditions tested in mice, we did not observe statistically significant increases in FD10 and TAMRA-D70 leakage for cCPEm+FUS^+MB^ at either PNP compared to the FUS^+MB^ treatment (Fig. 5A-D, Fig. 6J,K) 14.

### Staining for endogenous immunoglobulin provides a sensitive, complementary readout for blood-brain barrier opening

Given that neither FD10 nor TAMRA-D70 dextran leakage were detectable after the FUS^+MB^ and cCPEm+FUS^+MB^ treatments at the lower PNPs of 0.2 and 0.3 MPa (Fig. 6G,H,J,K), we next asked whether extravasation of endogenous IgG would be a more sensitive detection method, using brain sections and again including a sham control (Fig. 6C). Interestingly, endogenous IgG uptake was detectable at these low pressures, being prominent for most spots at 0.3 MPa (Fig. 6I,L, white arrows), and for fewer spots at the lower intensity of 0.2 MPa (Fig. 6I). Again, no uptake was found for the cCPEm group (Fig. 6F). The MFI values quantified for the sonicated spots in both the FUS^+MB^ and cCPEm+FUS^+MB^ group were significantly higher than those for the cCPEm and sham groups (*p<0.01*) (Fig. 6M). Together, the difference between detecting BBB opening via exogenously administered dextran and via endogenous IgG may be due to differences in detection sensitivities, and/or different uptake routes for the two types of cargoes.

## Discussion

Given that the BBB presents a major hurdle to drug delivery, we here performed a multimodal analysis, exploring two strategies of transiently opening the BBB in wild-type mice, the intravenous injection of the claudin-5-binder cCPEm, a BBB-weakening molecule, and FUS^+MB^, a modality that facilitates both transcytoplasmic and paracellular transport, the latter assumed to be due to the separation of claudin-5 strands. We determined a PNP threshold of between 0.3 and 0.35 MPa for FUS^+MB^-mediated BBB opening for the leakage of intravenously administered 10 kDa and 70 kDa dextran (FD10 and TAMRA-D70). However, when we explored the leakage of endogenous IgG (sized 150 kDa), we were able to lower the detection threshold for this cargo to a PNP of 0.2 MPa, possibly reflecting differences in sensitivity and the extent of labelling with the fluorophore, or a different mode of brain uptake of the two cargoes. Here, size is not the only determining factor, as IgGs have a unique shape and a hinge region providing flexibility that sets them apart from cargoes such as dextrans. Moreover, they contain a constant region (Fc) and many cell-types including brain endothelial cells express Fc receptors with which antibodies may engage. Therefore, IgGs may use Fc receptors for their ultrasound-mediated uptake and passage into the interstitial space. Furthermore, we explored cCPEm alone and as a pre-treatment preceding FUS^+MB^ at 0.2 and 0.3 MPa and found, different from our *in vitro* model 8, 14, no leakage for cCPEm on its own, and no augmented leakage for the combination treatment compared to FUS^+MB^. Finally, we used PCD to correlate dextran leakage with harmonic and broadband emissions.

Targeting components of the BBB and in particular TJ proteins such as claudin-5 is an emerging strategy to enhance cerebral drug delivery 28. Dithmer and colleagues designed peptides for the ECL1 domain of claudin-5, with mC5C2 displaying nanomolar affinity and achieving increased paracellular transport of dextrans up to a size of 40 kDa across brain endothelial and claudin-5-transfected epithelial cell monolayers 29. The protein cCPEm, on the other hand, is a variant derived from an enterotoxin. This variant targets claudin-5's ECL2, achieving leakage of 10 kDa dextran in zebrafish larvae 7, 8. We had previously shown in a claudin-5-overexpressing BBB-like MDCK (eGFP-hCldn5-MDCK II) *in vitro* system, that cCPEm weakens the barrier, and that pre-treatment with cCPEm lowers the PNP required for FUS^+MB^-mediated opening as revealed by reductions in TEER measurements and increased 40 kDa dextran leakage 14. Why cCPEm was neither effective in BBB opening in mice in the current study when used on its own nor has been augmenting BBB opening when used prior to FUS^+MB^ remains to be determined. We cannot rule out the possibility that cCPEm, were it administered at a higher, potentially toxic dose or via repeat treatments over extended periods, would achieve BBB weakening when used on its own in mice. However, our dose of cCPEm was four-fold higher than that of an analogue used in a study in mice addressing toxicity 30. We also did not reveal cCPEm-augmented BBB opening by IgG leakage, that had a lower detection threshold than the two dextrans. What may have contributed to this outcome is the narrow therapeutic window of drugs targeting claudin-5 31. Furthermore, Suzuki and co-workers have evaluated the antigenicity profile of cCPE, an analogue of cCPEm, in mice, showing that repeated nasal administrations (up to 6 times) of cCPE at 2 mg/kg resulted in an elevation of cCPE-specific serum IgG 32. This suggests potential antigenicity when applying cCPEm in mice; however, in general, small peptides display low antigenicity. Together, this finding illustrates the suitability of using endogenous IgG for detecting BBB leakage at low pressures, but also highlights the challenges in using BBB-weakening molecules such as cCPEm for an effective BBB opening, by taking *in vitro* findings to an *in vivo* model.

A limitation of our study is the dose of intravenously administered fluorescently tagged dextran, although the concentrations used by us have been informed by earlier studies 33, 34. Regarding the differences between dextran and endogenous IgG uptake, it is possible that different modes of transport are being used for the two types of cargoes which are differentially affected by cCPEm and FUS^+MB^ treatment. We have previously reported that large molecules cross the BBB via caveolin-mediated transcytosis following FUS^+MB^ treatment 34. Furthermore, in the mice, the concentration of endogenous IgG is almost ten times higher than the peak concentration of the administered dextran 35. Also, whereas serum IgG is consistently being generated and maintained at a stable concentration in the circulation (and hence is available to enter the brain over a frame of many hours as the BBB slowly closes after FUS^+MB^ treatment), intravenously injected dextran is rapidly removed by renal excretion 36. Other possibilities are differences in fluorescent labelling and sensitivity of the staining methods and in the imaging analysis.

In our study, we also assessed acoustic emissions. Detection of broadband emission, particularly when conducted in small animals, has been typically associated with the extravasation of red blood cells, a safety concern prompting the need for the development of feedback controllers based on harmonic emission 24, 37-39. In these studies, the controller is used to decrease the PNP as a safety measure as soon as broadband emission is detected. Interestingly, in our study, the onset of broadband emission did not lead to tissue damage. In fact, the threshold for broadband emission was found to be lower than that for BBB opening detected by dextran leakage. At 0.25 MPa, despite broadband emission being detected at a low intensity, no dextran leakage was found, indicating that the threshold pressure for BBB opening detected with dextran (between 0.3 and 0.35 MPa) was higher than that for broadband emission (between 0.15 and 0.25 MPa). In addition, broadband emission showed a good correlation with the PNP up to a pressure of 0.7 MPa. Safe BBB opening was achieved at 0.35 MPa, and small bleeds were detected in only one spot out of a total of 20 sonication sites in the four mice treated at 0.45 MPa. Within this PNP range, broadband emission also correlated with dextran leakage. Harmonic emission showed a correlation with dextran leakage, but this correlation was statistically not significant, and there was a steeper MFI slope for the harmonic compared with broadband emission. Overall, these data suggest that the development of a feedback control based on broadband emission is feasible, allowing for using such a controller in future studies.

As discussed by us recently 3, the PNP threshold for BBB opening may be higher in large animals and humans compared to mice. In humans, the threshold was found to be between 0.7 and 0.8 MPa 40. In a follow-up study, 1.03 MPa was used to treat 9 patients every 2 weeks, repeated 7 times 41. In total, 63 sonications were performed, resulting in no tissue damage as revealed by FLAIR or T2* MRI sequences. Since in this human study, the transducer was implanted into the skull (with no skull attenuation), we consider the reported PNP to be highly reliable in determining the threshold for BBB opening in humans. Considering that the mechanical index reported in this study was above 0.6 41, we consider it also highly likely that broadband emission was generated under these conditions and hence, may not necessarily be associated with tissue damage.

The underlying assumption for developing harmonic-based feedback controllers is that harmonic emission is caused by microbubble oscillation, i.e., stable cavitation, whereas broadband emission is an indicator of microbubble collapse, termed inertial cavitation. However, observations of single microbubble dynamics revealed that such a binary classification of cavitation may be too simplistic 16. It has been shown that harmonic emission without broadband emission can be generated from a single microbubble collapsing periodically, termed 'stable-inertial' cavitation 42. In this regime, the microbubble collapse not only generates harmonics, but also sub- and ultra-harmonics as well as broadband emission. Harmonics develop at relatively lower PNPs, whereas sub- and ultra-harmonics develop at relatively higher PNPs 16, 43, 44, when a microbubble grows to assume larger sizes which results in an extended collapsing period. As for broadband emission, as an underlying mechanism, a jitter in the interval of detected shock waves has been suggested, which can be produced by the displacement of several microbubbles or the formation of a microbubble cloud due to fragmentation. Collectively, the data suggest that the source of harmonic emissions during BBB opening may be the periodic collapse of microbubbles, classified as inertial cavitation or, more specially, 'stable-inertial' cavitation. In addition, we note that sub-harmonic emissions are being detected during the BBB opening procedure with the ExAblate device termed “broadband in nature” 45, which reconciles the 'stable-inertial' cavitation concept.

One reason for the early detection of broadband emission in our study may be the higher sensitivity of our PCD detection system. While a 20 dB preamplifier is often used 38, 46-48, our detection system consisted of a 34 dB preamplifier. Its noise characteristics are low (10 µVpp), and furthermore, our detector is composed of piezoceramic material with superior sensitivity compared to PVDF polyvinylidene fluoride. Collectively, although a direct comparison between studies is challenging without having calibrated the PCD sensitivity, we would argue that our detection system has a particularly high sensitivity.

The observed differences in the broadband response can also be attributed to our ultrasound and microbubble parameters. We speculate that the use of a relatively fast PRF of 10 Hz, compared to 1 or 2 Hz used in other studies 24, 38, 39, 49 could alter the interaction of FUS and microbubbles significantly. 10 Hz PRF and 6 s sonication per spot were chosen given that these parameters achieve good BBB opening in mice 19, 36, 50. A 10 Hz PRF, compared to 1 or 2 Hz, shortens the microbubble replenishment time by a factor of 5 to 10, respectively. This could result in relatively lower microbubble concentrations for pulses except for the very first pulse, for which a longer replenishment time was provided while the transducer was moved from one sonication spot to the next. This may explain the observed 'intensity drop', given that for most spots, the intensity of the first pulse was notably higher than for the remainder of the pulses for a given sonication spot (Fig. 3B,C). Assuming an unaltered pressure field between pulses, the relative reduction in microbubble concentration is a likely explanation. Similar PCD responses to those reported by us have been reported in a study which used a PRF of 5 Hz 51. Overall, we demonstrated in our study that the use of a fast PRF of 10 Hz in combination with a short sonication duration of 6 seconds opened the BBB safely in a way that can be predicted based on the PCD response, and that in particular the broadband emission is a preferred component due to its robust baseline (Fig. 3E) and its wider intensity range compared to the harmonic emission when correlated with dextran leakage (Fig. 3G,H).

Another aspect for consideration is the role of microbubble concentrations in relation to the PNP threshold for broadband emission. An *in vitro* study demonstrated that the PNP threshold could be lowered when a larger capillary was sonicated which contains more microbubbles 52. Similarly, the presence of larger microbubbles (4-5 µm and 6-8 µm) 48 (i.e., a higher microbubble volume concentration 53) has been found to lower the PNP threshold for broadband emission and BBB opening in mice. In contrast, for smaller microbubbles (1-2 µm), the PNP threshold for broadband emission was the same as for BBB opening 48. Together with our data, this would indicate that broadband emission is not the consequence of tissue damage.

## Conclusion

We conclude that the histological detection of endogenous IgG is a potentially robust means for detecting BBB opening histologically, given that FUS^+MB^-induced BBB opening was detectable at a pressure ≥0.35 MPa when assessed for leakage of 10 and 70 kDa dextran, but at ≥0.2 MPa for uptake of endogenous IgG. Regarding the utilisation of cCPEm, our results indicate that further exploration of dosing parameters is required to realise the beneficial effects of a cCPEm+FUS^+MB^ combination treatment in mice, and that the translation of *in vitro* findings to an *in vivo* system remains challenging. Importantly, by passive cavitation detection, we found that broadband emission was correlated with the peak negative pressure (PNP) and with dextran leakage, indicating the possibility of using broadband emission for developing a feedback controller to monitor BBB opening. Together, our study highlights the challenges in developing combinatorial approaches to open the BBB and presents an additional IgG-based histological detection method for BBB opening.

## Materials and Methods

### Animal husbandry and ethics approval

The three-month-old C57BL/6J (wild-type) mice used in this study were housed in small groups in an environmentally controlled room at 23 ± 1.5 ºC with a 12 h light/12 h dark cycle and constant access to water and food. All animal experiments were conducted under the guidelines of the Australian Code of Practice for the Care and Use of Animals for Scientific Purposes and were approved by the Animal Ethics Committee of the University of Queensland (approval number QBI/554/17/NHMRC). The study is reported in accordance with ARRIVE guidelines.

### cCPEm generation and characterisation

GST-cCPEm (in short: cCPEm) was generated as previously reported 14. Briefly, BL21(DE3) *E. coli* (New England Biolabs, Ipswich, Massachusetts, United States, cat. #C25271) were transformed with plasmid pGEX-4T1-cCPEm encoding the carboxy-terminal domain of CPE (aa 194-319) together with two mutations, Y306W and S313H. The bacteria were grown to OD_600_ ~ 0.6, before adding isopropyl-β-D-thiogalactopyranoside (IPTG, Meridian Bioscience, Cincinnati, Ohio, United States, cat. #BIO-37036) to a final concentration of 1 mM to induce gene expression. The bacteria were then harvested by centrifugation after 6 h at 30 ºC with constant vigorous shaking. The bacterial pellet was lysed after 20 min incubation on ice by intermittent sonication using the Sonics Vibra-Cell VCX130 sonicator (Newtown, Connecticut, United States) in lysis buffer (25 mM tris-HCl, pH 8; 150 mM NaCl, 5 µM 1,4-dithiothreitol) (DTT) containing protease inhibitors (Complete Mini, EDTA-free; Roche Applied Science, Mannheim, Germany), 10 U/ml benzonase nuclease (Sigma-Aldrich, St. Louis, Missouri, United States, cat. #E1014) and 100 µg/ml lysozyme (Amresco, Solon, Ohio, United States, cat. #0663-10G). After sonication, the bacterial lysate was centrifugated at 20,000 g for 30 min at 4 ºC, and the collected supernatant was filtered through a 0.22 µm syringe filter (Millipore, cat. #SLGP033RS, Billerica, Massachusetts, United States). The filtered supernatant of the bacterial lysate was then purified by affinity chromatography using an automated Profinia Protein Purification system (Bio-Rad, Hercules, California, United States), followed by size-exclusion chromatography using a Superdex 200 Increase 10/300 GL column (GE Healthcare, cat. #28990944) with an ÄKTApurifier chromatography system (GE Healthcare) in 1× phosphate-buffered saline (PBS) containing 1 mM DTT. To identify the cCPEm fractions, the A280 peak fractions were collected and assayed for GST expression by immunoblotting with an anti-GST antibody (Proteintech, cat. #66001-2-1g). The cCPEm-containing fractions were pooled and further concentrated to a final concentration of 6 mg/ml using centrifugal filters (Amicon ^®^Ultra-4, Merck Millipore, cat. #UFC801024) with a molecular cut-off of 10 kDa. For *in vivo* applications, the protein was further purified using PierceTM High-Capacity Endotoxin removal Spin Columns (ThermoFisher cat. # 88274). Aliquots of cCPEm were stored at -80 ºC until use.

### Fluorescently labelled dextran

Fluorescently labelled dextrans were used as model drugs for visually tracing BBB opening. Dextran labelled with fluorescein isothiocyanate (FD10) has a molecular weight of 10 kDa (Invitrogen, cat. #D1820) and a hydrodynamic radius (R_H_) of ~2.3 nm. Tetramethylrhodamine isothiocyanate labelled-dextran (TAMRA-D70) has a molecular weight of 70 kDa (Sigma-Aldrich, cat. #T1162) and a hydrodynamic radius (R_H_) of ~6.5 nm. FD10 and TAMRA-D70 were diluted to a stock concentration of 20 mg/ml and 50 mg/ml in PBS before use, respectively.

### Custom-designed microbubbles

Custom-designed microbubbles comprising a phospholipid shell (DSPC and DSPE-PEG2000) and octafluoropropane gas (C_3_F_8_) core were generated as previously reported (Fig. 1C) 14. On the day of experimentation, prior to systemic administration, microbubbles were brought to room temperature (RT) and activated by agitation in a dental amalgamator at 4,000 rpm for 45 seconds followed by a characterization using a Multisizer 4e Coulter Counter (Beckman Coulter) to ensure that the microbubble concentrations and size distribution were consistent across treatments.

### Ultrasound equipment and cavitation measurement

An integrated focused ultrasound system (Therapy Imaging Probe System, TIPS, Philips Research, centre frequency of 1 MHz) was used 19. The system consists of an annular array transducer (80 mm radius of curvature, housed in an 80 mm spherical shell, with a central opening of 31 mm in diameter), a 3D positioning system, and a programmable motorized system to move the ultrasound focus in the x and y planes. A coupler mounted to the transducer was filled with degassed water and placed above the head of the mouse using a 1 mm film of ultrasound gel for coupling. The transducer generated a focus that was 1.5 mm × 12 mm in the transverse and axial planes, respectively. The motorized positioning system moved the focus of the transducer array, allowing for a 1.5 mm spacing between individual sites of sonication.

Sonication with the TIPS system induces cavitation of the injected microbubbles. Cavitation activities were simultaneously monitored using a custom-built passive cavitation detection system comprised of an unfocused 1/4"diameter single-element transducer (3.5 MHz, V384-N-SU; Olympus NDT Inc., MA, USA) as PCD, a 34 dB pre-amplifier (Model 5662; Olympus NDT Inc.), and a 16-bit digitiser card (M4i.4421; Spectrum Instrumentation GmbH, Grosshansdorf, Germany) sampling at 31.25 MHz. The recorded PCD signals were processed off-line as reported previously using a custom developed MATLAB (MathWorks Inc., MA, USA) script 50. Higher harmonic (HH) and broadband (BB) emissions were extracted in the frequency domain with the following frequency band: (with *f_0_* = 1 MHz):

• HH: 4*f_0_* - 30 kHz ≤ BW ≤ 4*f_0_* + 30 kHz and 5*f_0_* - 30 kHz ≤ BW ≤ 5*f_0_* + 30 kHz
• BB: 3*f_0_* + 30 kHz ≤ BW ≤ 4*f_0_* - 30 kHz and 4*f_0_* + 30 kHz ≤ BW ≤ 5*f_0_* - 30 kHz

where *f_0_* = 1 MHz. A 30 kHz interval was empirically determined. Lastly, each component's intensity was calculated using Parseval's theorem as following:



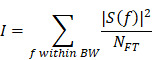



where *S(f)* and *N_FT_* denotes the Fourier transform of the PCD signals and the number of frequency bins in the spectrum, respectively.

### FUS^+MB^ and cCPEm treatments

The mice were assessed during daytime in two cohorts (Fig. 1A) subjected to FUS^+MB^ over a range of PNPs, cCPEm, cCPEm combined with FUS^+MB^ at different pressures (cCPEm+FUS^+MB^), and sham. For cohort 1, for anaesthesia, ketamine (90 mg/kg) and xylazine (6 mg/kg) diluted in 0.9% NaCl solution were injected intraperitoneally, using a 27-gauge needle. For the entire cohort 2, as mandated by the animal ethics committee to account for the extended treatment duration, isoflurane was first used for cCPEm administration (with and without FUS^+MB^) at the time point -2.5 hours, such that the mice could be recovered quickly before treating them at time point 0 with ketamine and xylazine for sonication.

After anaesthesia, the hair of the mouse's head was removed with clippers and hair removal cream. A catheter was inserted into the tail vein of the mouse consisting of a PE20 tubing attached to a 30-gauge needle. The mice were then positioned in a stereotaxic frame under the TIPS transducer. To visualize BBB opening, first, FD10 was injected intravenously at a dose of 0.04 mg/gbw and TAMRA-D70 at 0.05 mg/gbw via the vein catheter. Immediately thereafter, the microbubbles were bolus-injected by hand at a dose of 1 µl/gbw over a period of ~ 3 seconds, using a canula (Fig. 1A). This was always followed by a 50-60 µl saline flush. Additionally, for cohort 1, two rows in one hemisphere were sonicated over a period of ~ 100 seconds, followed by pausing for 5 minutes to ensure complete microbubble clearance. This was followed by a second bolus injection of the microbubbles, factoring in the dynamics of microbubble concentration and replenishment reported previously in mice 54. Then, the remaining two rows in the second hemisphere were sonicated over a period of again ~ 100 seconds. The following ultrasound parameters were used: 1 MHz frequency, 10 Hz PRF (pulse repetition frequency), 10% duty cycle, 10 ms PL (pulse length), and a 6 s sonication time per spot, using a range of PNPs. At the conclusion of the treatments, mice were placed in a warm incubator and monitored until recovery. After 1.5 hours, the mice were sacrificed and transcardially perfused with PBS at room temperature.

### Mouse brain imaging and mouse tissue imaging

The PBS-perfused mouse brains were then collected and immediately imaged in a Bruker *In Vivo* MS FX Pro optimal imaging system with a 480 or 540 excitation filter for detecting FD10 and a 540 or 570 nm emission filter for detecting TAMRA-D70, respectively. Then, the brains were immersion-fixed in 4% paraformaldehyde (PFA) for 24 hours, after which the brains were sectioned at 100 µm thickness using a vibratome (Leica), and approximately 35~40 sections were obtained per mouse. Starting at the top, the third section from the top was chosen, and then every sixth section, up to a total of five sections for each mouse. The selected sections were mounted on slides and imaged at a 10 × magnification using an upright epifluorescence Axio Imager Azure microscope. To quantify the uptake of dextrans, a region of interest (Fig. 2A), corresponding to the sonication spot with the maximal fluorescence signal intensity for a given section was chosen and subjected to analysis using ImageJ.

### Immunoglobulin staining

The selected sections were blocked in 10% w/v bovine serum albumin (BSA; Bovogen Biologicals, cat. #BSAS 0.1) and 5% goat serum for 1 h on a rocker at RT, followed by permeabilization in 0.1% Triton X-100 (Sigma-Aldrich) in blocking buffer for 30 min. The sections were then incubated with an Alexa Fluor™ 647-coupled goat anti-mouse IgG(H+L) antibody (Invitrogen, cat. #A28181, diluted 1:1,000) overnight on a rocker at 4ºC. The sections were washed three times via PBS, mounted on slides, and imaged at a 10 × magnification using an upright epifluorescence Axio Imager Azure microscope. A region of interest, corresponding to one sonication spot, was selected to quantify the extravasation of endogenous IgG using ImageJ (Fig. 6).

### Statistical analysis

Statistical analysis was done with GraphPad Prism 9.5.1 (San Diego, CA) using a one-way or two-way ANOVA with Tukey's multiple comparison test. Unless stated otherwise, data are presented as mean ± SEM. For all comparisons, the level of significance was set at *p< 0.05. Schematic illustrations were generated with bioRender.com.

## Figures and Tables

**Table 1:** ** BBB opening as a function of the acoustic pressure and observed by brain uptake of two fluorescently labelled dextrans, FD10 (10 kDa) and TAMRA-D70 (70 kDa).** 5 sonications spots in 4 mice quantifying 5 sections (cohort 1) each adding up to a maximum of 100 spots per experimental condition.

PNP (MPa)	Dextran		Dextran leakage (BBB opening spots out of total sonication spots)	
	Mw (kDa)	RH (nm)		
0.15	10	2.3	-	(0/100)
	70	6.5	-	(0/100)
0.25	10	2.3	-	(0/100)
	70	6.5	-	(0/100)
0.35	10	2.3	++	(49/100)
	70	6.5	+	(47/100)
0.45	10	2.3	+++	(39/100)
	70	6.5	+++	(39/100)

**Table 2:** List of intensities of harmonic and broadband emissions.

PNP (MPa)		0.15	0.25	0.35	0.45	0.7
**Harmonic (dB)**	Cavitation	55.4 ± 6.0	63.3 ± 4.4	65.3 ± 3.8	67.8 ± 4.3	73.9 ± 5.6
	Baseline	26.2 ± 1.0	28.1 ± 2.0	33.9 ± 2.6	32.8 ± 3.1	29.0 ±1.6
	Enhancement	29.1 ± 2.6	35.0 ± 3.8	31.2 ± 2.7	34.5 ± 1.5	45.0 ± 3.6
**Broadband (dB)**	Cavitation	35.8 ± 0.6	39.4 ± 2.1	42.4 ± 3.1	49.0 ± 5.0	57.0 ± 7.8
	Baseline	35.1 ± 0.2	35.3 ± 0.1	35.3 ± 0.2	35.2 ± 0.1	34.5 ± 0.1
	Enhancement	0.7 ± 0.3	4.2 ± 0.8	7.2 ± 0.5	13.8 ± 3.0	22.5 ± 5.2

## Abbreviations

AJ: adherens junction
BBB: blood-brain barrier
bEND.3: murine brain vascular endothelial cell
cCPEm: truncated form of clostridium perfringens enterotoxin
C_3_F_8_: octafluoropropane gas
CNS: central nervous system
DTT: 1,4-dithiothreitol
ECL: extracellular loop domain
FD10: 10 kDa dextran labelled with fluorescein isothiocyanate
FUS: low-intensity focused ultrasound
FUS^+MB^: focused ultrasound combined with intravenously injected microbubbles
IgG: immunoglobulin
IPTG: isopropy1-β-D-thiogalactopyranoside
MFI: mean fluorescence intensity
NVU: neurovascular unit
PC: personal computer
PCD: passive cavitation detection
PL: pulse length
PNP: peak negative pressure
PRF: pulse repetition frequency
RF: radio frequency
ROI: region of interest
RT: room temperature
TAMRA-D70: 70 kDa dextran labelled with tetramethylrhodamine isothiocyanate
TEER: transendothelial electrical resistance
TJ: tight junction
TIPS: therapy imaging probe system
